# Salvage surgeries for splanchnic artery aneurysms after failed endovascular therapy: case series

**DOI:** 10.1097/JS9.0000000000000442

**Published:** 2023-05-18

**Authors:** Yi-Chun Lin, Tzu-Chi Liao, Chien-Te Lin, Long-Bin Jeng, Horng-Ren Yang, Chung-Ho Hsu, Wei-Ching Lin, Ching-Feng Wu, Chun-Chieh Yeh

**Affiliations:** a School of Medicine, China Medical University, Taichung; b Department of Medical Education, Chang Gung Memorial Hospital, Kaohsiung; c Department of Surgery, China Medical University Hospital, China Medical University, Taichung; d Department of Medicine, China Medical University Hospital, China Medical University, Taichung; e Department of Medical Imaging, China Medical University Hospital, Taichung; f Department of Biomedical Imaging and Radiological Science, School of Medicine, China Medical University, Taichung; g Department of Surgery, Asian University Hospital, Taichung, Taiwan

**Keywords:** case series, chronic pancreatitis, failed endovascular therapy, false aneurysm, mass effect, salvage surgery

## Abstract

Splanchnic arterial aneurysms are a rare but potentially lethal disease with a mortality rate of more than 10% after rupture. Endovascular therapy is the first-line treatment for splanchnic aneurysms. However, appropriate management for splanchnic aneurysms after failed endovascular therapy remained inconclusive.

**Materials and methods:**

A retrospective review was performed for consecutive patients (from 2019 to 2022) who underwent salvage surgeries for splanchnic artery aneurysms following failed endovascular therapy. The authors defined failed endovascular therapy as the technical infeasibility to apply endovascular therapy, the incomplete exclusion of the aneurysm, or the incomplete resolution of preoperative aneurysm-associated complications. Salvage operations included aneurysmectomy with vascular reconstruction and partial aneurysmectomy with directly closing of bleeders from the intraluminal space of the aneurysms.

**Results:**

Seventy-three patients received endovascular therapies for splanchnic aneurysms, and 13 failed endovascular trials. The authors performed salvage surgeries for five patients and enrolled them in this study, including four false aneurysms of the celiac or superior mesenteric arteries and a true aneurysm of the common hepatic artery. The causes of failed endovascular therapy included coil migration, insufficient space for safely deploying the covered stent, a persistent mass effect from the postembolized aneurysm, or infeasibility for catheter cannulation. The mean hospital stay was nine days (mean±SD, 8.8±1.6 days), with no one suffering 90-day surgical morbidity and mortality, and all patients getting symptoms improvement. During the follow-up period (mean±SD, 24±10 months), one patient suffered a small residual asymptomatic celiac artery aneurysm (8 mm in diameter) and was treated conservatively due to underlying liver cirrhosis.

**Conclusion:**

Surgical management is a feasible, effective, and safe alternative for splanchnic aneurysms after failed endovascular therapy.

## Introduction

Highlights

- Salvage surgery for splanchnic aneurysm after a failed endovascular trial is feasible.
- The way of salvage surgery is decided by the anatomy of the aneurysm and the surrounding environment.
- Intraluminally closing the vascular orifice of the splanchnic aneurysm is suggested in a hostile abdomen.

Splanchnic arterial aneurysms are rare but can be a potentially lethal disease. The incidence of splanchnic arterial aneurysms is 0.1–2%^1,2^. Approximately 25% of splanchnic arterial aneurysms initially presented as rupture, and the mortality rate after rupture is more than 10%, highlighting the importance of timely diagnosis and adequate management^2–5^.

Timely diagnosis of true or false aneurysms by history and images was essential to prompt appropriate management because of the risk of rupture. Ruptured splanchnic true aneurysms mainly occur when their diameter is more than 2 cm^2,3,6^. Besides, gastroduodenal artery (GDA), superior mesenteric artery (SMA), and pancreaticoduodenal artery true aneurysms differ from others (e.g., splenic arterial aneurysm) by a higher risk of rupture regardless of the size^5^. Similarly, false splanchnic aneurysms have a significant risk of rupture, irrespective of size^5,7^. Thus, selected true splanchnic aneurysms (e.g., any GDA, SMA, pancreaticoduodenal artery aneurysms, aneurysm greater than 2 cm, or symptomatic aneurysms) and false aneurysms may warrant timely intervention to avoid life-threatening rupture^2,3,5,6^.

Previous studies reported that their strategies for treating splanchnic aneurysms varied depending on the anatomical location, size, pathophysiological processes, collateral flow, true or false aneurysm, ruptured or intact aneurysms, and hemodynamic status^2,3,5,6,8^. Because of its minimally invasive characteristics, endovascular therapy has been the first-line treatment for splanchnic aneurysms; however, around 10% of cases underwent failed endovascular therapy due to incomplete exclusion of the aneurysm or infeasibility in catheterization^6^. Tortuous parent vessels, irregularity of the aneurysm, and inadequate proximal or distal landing zone predispose to failed endovascular therapy in splanchnic aneurysms^2^. Surgical intervention is the previously definitive and currently alternative treatment for splanchnic aneurysms with higher morbidity and comparable mortality but less reintervention rate than endovascular therapy^1,9,10^. However, only some previous studies have comprehensively described surgical details.

The unique anatomic position of the splanchnic aneurysm makes us have to take special consideration for its management. For example, a larger-sized splanchnic aneurysm may cause compression effects such as pancreatitis, obstructive jaundice, or intestinal obstruction^11–13^. Nonetheless, aneurysm-associated mass effects were less discussed before. In addition, tortuous vascular anatomy makes endovascular therapy for splanchnic aneurysms challenging and unexpectedly risky (e.g., nontarget organ ischemia). Most studies concerning splanchnic aneurysms were published from the perspectives of interventional radiologists, cardiovascular physicians, or cardiovascular surgeons. Hence, from the general surgeon’s perspective, we would like to share our experience managing splanchnic aneurysms, particularly after failed endovascular therapy or aneurysms with associated mass effects. This educational case series may impact and guide the future clinical practice of surgeons while they fail endovascular trials for the splanchnic aneurysm.

## Materials and methods

**Figure 1:**
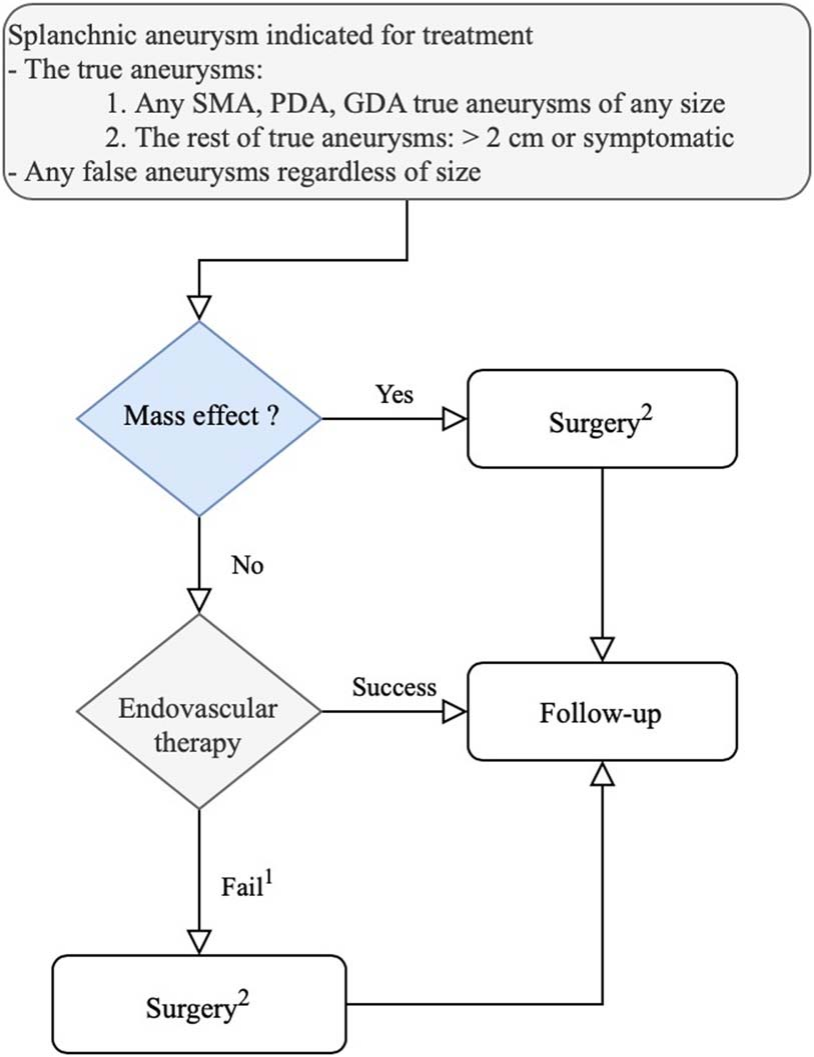
The decision-making flow chart for splanchnic aneurysms. ^1^Failed endovascular therapy is defined as infeasibility to apply endovascular therapy, incomplete exclusion of the aneurysm, or the incomplete resolution of preoperative aneurysm-associated complications. ^2^Surgery includes one of the following three strategies: (1) aneurysmectomy along with the involved organ if the aneurysm is located within the parenchyma of solid organs (2) aneurysmectomy with vascular reconstruction if the aneurysm outside the solid organ and from nonexpendable feeding vessels (3) partial aneurysmectomy with direct closing of the feeding vessel orifice if the aneurysm occurs in the hostile abdomen or from expendable feeding vessels.

Between 2019 and 2022, this retrospective case series consecutively enrolled patients who received salvage surgeries for splanchnic arterial aneurysms after failed endovascular therapy in a tertiary referral teaching hospital. The splanchnic aneurysms were mainly diagnosed by computed tomography (CT). The interventional radiologist or cardiovascular physician will always be consulted first for endovascular trials to treat a newly diagnosed splanchnic artery aneurysm. The definition of failed endovascular therapy includes technical infeasibility to apply endovascular therapy, incomplete exclusion of the aneurysm, or the incomplete resolution of preoperative aneurysm-associated complications. We offered tailored salvage surgeries for splanchnic artery aneurysms after failed endovascular trials (Fig. 1). Perioperative information (e.g., symptoms, comorbidity, risk factors, and anatomical characteristics of the aneurysms, the causes of failed endovascular therapy, surgical details, postoperative complications, and outcomes) were retrospectively collected by reviewing medical records. We achieved the deidentification of patient information by the expert determination method. Written informed consent for publication was obtained while the patient visited the outpatient clinic. The ethical issues of this study had been reviewed and approved by the local institutional review board (IRB) (IRB no.: CMUH110-REC1-182) on 3 January 2022 and registered in a publicly accessible database (Registered ClinicalTrials.gov ID: NCT05727956). This case series has been reported in line with the PROCESS Guideline^14^, Supplemental Digital Content 1, http://links.lww.com/JS9/A504.

### Salvage surgical technique

Suppose the splanchnic aneurysm was within the parenchyma of solid organs; in that case, aneurysms and involved parenchyma or organs would be resected (e.g., aneurysmectomy with splenectomy for the aneurysm close to the spleen). Suppose the aneurysm was located outside the parenchyma of solid organs and from nonexpendable feeding vessels; the aneurysm’s proximal and distal vascular ends would be controlled first, followed by aneurysmectomy and vascular reconstruction. Nevertheless, when encountering a hostile abdomen (e.g., false aneurysms secondary to chronic pancreatitis) or aneurysms from expendable feeding vessels, a partial aneurysmectomy would be performed first, followed by closing the orifice of feeding vessels from the intraluminal space of the aneurysm. The decision-making flowchart (Fig. 1) and direct repairing of feeding vessels from the intraluminal approach in the hostile abdomen were first proposed for patients who failed endovascular trials for splanchnic aneurysms.

The details of the direct repairing of feeding vessels were as follows: After the surgical and anesthetic team were well-prepared for bleeding control and rapid blood transfusion, the aneurysm would be incised, followed by compressing the major bleeder by the fingertips and immediately closing the orifice from the intraluminal space with polypropylene suturing. In the saccular aneurysm, the orifice would be sutured carefully to avoid suturing the opposite vascular wall so that the patency of the lumen could still be maintained. In the fusiform aneurysm, the two bleeders could be sutured from the intraluminal space after opening the aneurysm without compromising organ perfusion due to abundant collateral circulation in the splanchnic vascular system. However, in some nonexpendable vessels (e.g., SMA trunk, proper hepatic artery), aneurysmectomy with the restoration of blood flow by vascular reconstruction was still needed. The thrombosed hematoma would be evacuated, and the dead space within the aneurysm would be packed with an omentum to avoid infected fluid accumulation. If the aneurysm was embedded within the pancreas region, the edge of the aneurysmal wall would be secured by running suturing to avoid pancreas juice leakage. All surgical procedures were performed by a surgical team consisting of a single well-trained general surgeon and a cardiovascular surgeon. When patients were discharged, we would schedule outpatient clinic visits every 3 months and CT scans to detect recurrent aneurysms every 6–12 months. We would particularly educate patients to control hypertension through medication and lifestyle adjustments.

## Results

Seventy-three patients received endovascular therapies for splanchnic aneurysms between 2019 and 2022 in our hospital. Thirteen of 73 patients (18%) failed endovascular therapies. Seven of 21 cases (33%) failed elective endovascular treatments, and six of 52 cases (12%) failed emergent endovascular trials. Ten of them received salvage surgeries after failed endovascular therapies. Only five of them were treated by the authors and enrolled in this study. All detailed information (e.g., location and diameter of aneurysms, causes of failed endovascular trials) of these 73 patients were collected retrospectively (SDC, Table 1, Supplemental Digital Content 2, http://links.lww.com/JS9/A505). The size of the aneurysms undergoing failed endovascular attempts varied significantly (mean±SD, 38.3±40.7 mm), and only 4 of 13 cases (31%) were giant aneurysms (i.e., diameter larger than 40 mm). The size, the anatomic locations of the aneurysm, and the patient’s hemodynamic status all may account for the failure of endovascular therapies (SDC, Table 1, Supplemental Digital Content 2, http://links.lww.com/JS9/A505).

This study included five adults ( 3 males, 58.2±11.9 years) (Table 1). Four of the five lesions were false aneurysms of the celiac artery, SMA, and dorsal pancreatic artery. One case was a true aneurysm of the common hepatic artery involving bifurcation of the GDA and proper hepatic artery. The mean diameter of the aneurysms was 64 mm (mean±SD, 64.4±57.2 mm). Four patients also presented with abdominal fullness, jaundice, or abdominal pain. In the asymptomatic patient, the lesion was detected by sonography incidentally while surveying atorvastatin-related hepatitis.

**Table 1:** Demographics.

Case	1	2	3	4	5
Age	47	59	65	74	47
Sex	Male	Female	Male	Female	Male
TRUE Or FALSE aneurysm	FALSE	TRUE	FALSE	FALSE	FALSE
Etiology	Chronic pancreatitis	Idiopathic	Chronic pancreatitis	Idiopathic	Hypertension
Initial presentation	Abdominal fullness	Asymptomatic	Jaundice & Abdominal fullness	Abdominal pain and fullness	Epigastralgia and back pain
Location (artery)	Celiac	Common hepatic	Superior mesenteric	Dorsal pancreatic	Celiac
Largest diameter (mm)	60	30	60	160	12
Causes of failed endovascular therapy	- Tortuous vessels - Insufficient landing zone	- Tortuous vessels - Risk of occluding the right hepatic artery after deploying stent	- Coils migration - Risk of losing nearby SMA branches after deploying stent	- Coils migration -Persistent mass effect	- Difficulty in catheter cannulation
Surgical therapy	Partial aneurysmectomy with direct repair	Aneurysmectomy with end-to-end anastomosis	Partial aneurysmectomy with direct repair	Partial aneurysmectomy with direct repair	Aneurysmectomy with vascular reconstruction by a jumping graft
Outcome	Partial resolution with residual false aneurysm 8×8 mm	Complete resolution	Complete resolution	Complete resolution	Complete resolution
Follow-up (months)	39	24	24	22	11

Two patients failed in endovascular therapy due to incomplete exclusion of aneurysms after coil migration, and one of them also suffered incomplete resolution of aneurysm-associated compression signs. The other three patients failed endovascular trials due to the tortuous vascular anatomy, an insufficient landing zone, infeasibility for catheter cannulation, and an increased risk of occluding the nearby arteries if deploying the covered stent. After failed endovascular therapies, all five cases underwent salvage surgeries.

As for the types of salvage surgeries, only one patient underwent a complete aneurysmectomy with vascular anastomosis for the true aneurysm at the bifurcation of the proper hepatic artery and GDA. One patient received an aneurysectomy with vascular reconstruction using a great saphenous vein as a jump graft. The other three patients underwent partial aneurysmectomy, followed by direct suturing bleeders of the feeding vessels via an intraluminal approach. The average length of hospital stay was 9 days (mean±SD, 8.8±1.6 days), with no one suffering 90-day surgical morbidity and mortality.

All patients were kept from follow-up and monitored for hypertension at outpatient clinic visiting. Four out of five patients did not have recurrent aneurysms in the follow-up time (mean±SD, 24±10 months). However, only one case had an asymptomatic remaining false aneurysm (8 mm in diameter, Clavien–Dindo Classification grade 1). He was treated with well-controlling hypertension only due to the severe underlying comorbidity. (alcoholic liver cirrhosis, Child B). No one needed repeated invasive interventions.

## Case presentation

### Case_1- Celiac artery false aneurysm

**Figure 2:**
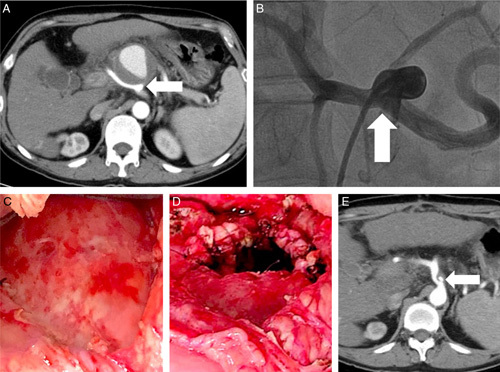
Celiac trunk false aneurysm (A) CT revealed an aneurysm from the celiac trunk (arrow). (B) The angiography showed a false aneurysm about 6 cm in diameter arising from the bifurcation of the celiac trunk(arrow). (C) The false aneurysm was about 7 cm in diameter, and challenging to control the feeding vessels from the outside of the aneurysm due to chronic pancreatitis-related dense adhesion. (D) We opened the aneurysm and directly sutured the bleeder from the inner lumen of the aneurysm. The rim of the false aneurysm cavity was oversewed for hemostasis and the prevention of pancreas leakage. The cavity was packed with an omentum patch to avoid re-accumulated infected fluid. (E) Follow-up CT showed a remaining small false aneurysm 8 mm in diameter (arrow) six months after the surgery.

A 47-year-old man with alcoholism-related liver cirrhosis and chronic pancreatitis suffered abdominal fullness for 1 week. The biochemical tests showed mildly rising liver function test (aspartate aminotransferase 102 U/l, total bilirubin 28.91 μmol/l, direct bilirubin 16.08 μmol/l). Abdominal CT demonstrated a celiac artery false aneurysm (Fig. 2A), and angiography showed a false aneurysm 6 cm in diameter arising from the bifurcation of the celiac artery (Fig. 2B).

He failed the endovascular trial because of tortuous vascular anatomy and an insufficient landing zone for deploying the covered stent. Then, a salvage laparotomy was performed for partial aneurysmectomy, directly repairing of the neck of the false aneurysm and running suturing around the edge of the open aneurysmal wall to avoid pancreas leakage (Fig. 2C, D). After 6 months, abdominal CT showed a remaining small false aneurysm around 8 mm in diameter (Fig. 2E). Considering the underlying decompensated liver cirrhosis, he chose conservative treatment by well-controlling blood pressure, and the aneurysm did not change its size for the postoperative 39 months.

### Case_2- Common hepatic artery true aneurysm

**Figure 3:**
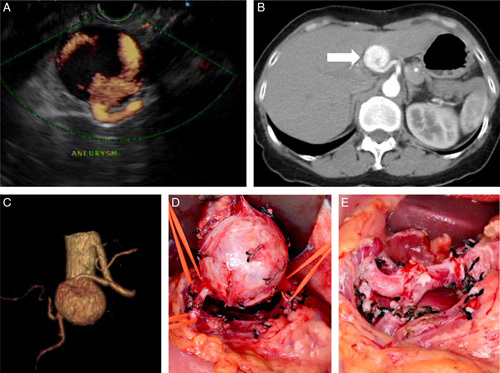
Common hepatic artery true aneurysm (A) Endoscopic ultrasonography showed a common hepatic artery aneurysm (3 cm in diameter). (B, C) The CT scan and CT angiography revealed an aneurysm located between the junction of the common hepatic artery, gastroduodenal artery, and proper hepatic artery. (D) The three feeding vessels of the aneurysm were dissected and controlled individually. (E) After excising the aneurysm, we did end-to-end vascular reconstruction between the common hepatic artery and the common orifice of the proper hepatic artery and the gastroduodenal artery.

A 59-year-old woman with type I diabetes mellitus and dyslipidemia was accidentally diagnosed with a pancreatic head mass lesion by abdominal sonography while surveying atorvastatin-related hepatitis. Endoscopic ultrasonography showed an aneurysm 3 cm in diameter connecting with the common hepatic artery (Fig. 3A). The CT scan and CT angiography revealed an aneurysm located between the bifurcation of the proper hepatic artery and GDA (Fig. 3B and C). Endovascular therapy failed because of the tortuous vascular anatomy and the risk of occluding the right hepatic artery if deploying the covered stent. Thus, a salvage laparotomy was performed to resect the aneurysm, followed by an end-to-end anastomosis between the common hepatic artery and the common orifice of the proper hepatic artery and GDA (Fig. 3D, E). No more recurrent aneurysm was observed in a 24-month follow-up.

### Case_3- Superior mesenteric artery false aneurysm

**Figure 4:**
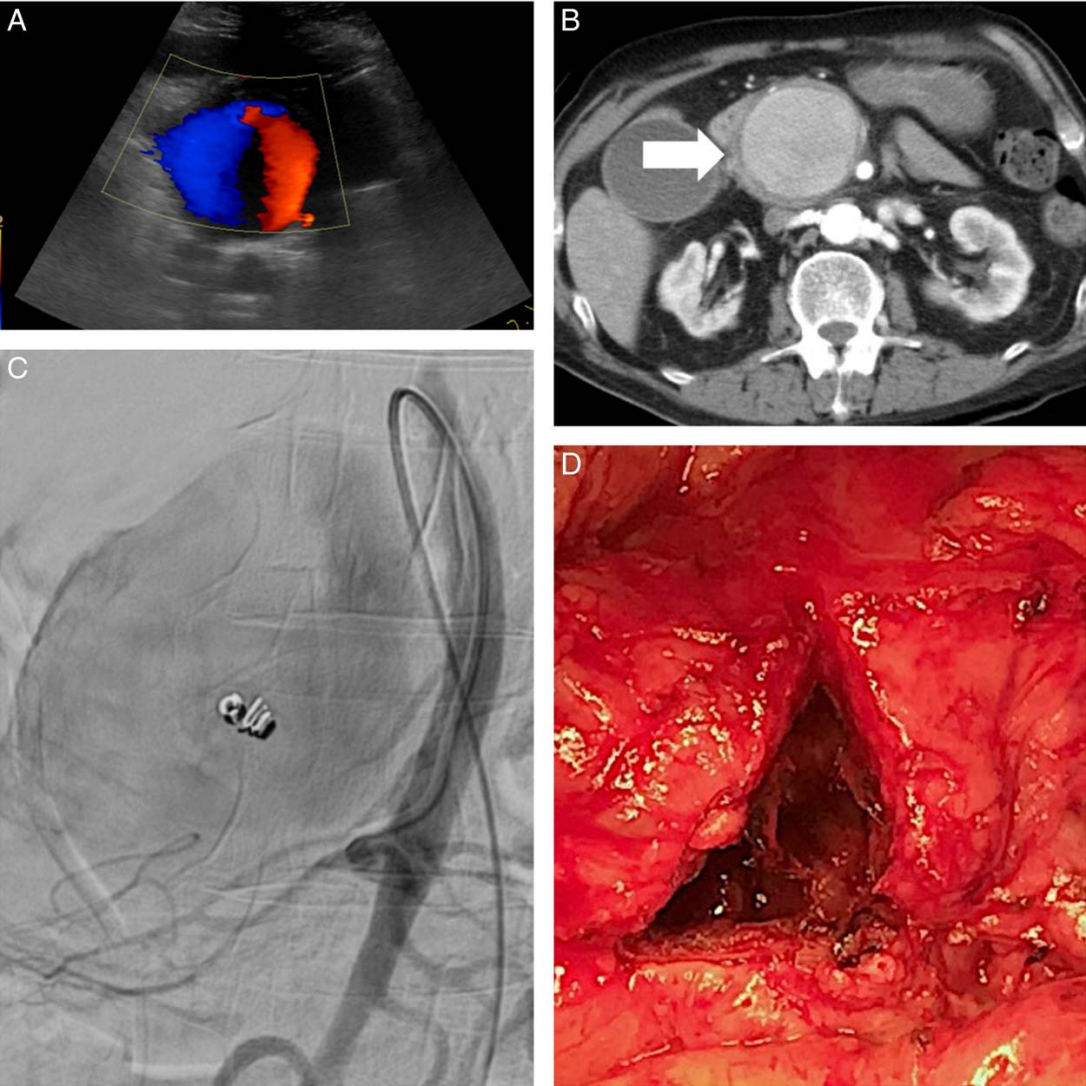
Peri-pancreatic false aneurysm (A) Abdominal sonography showed a cystic lesion with a ‘ying-yang’ sign that signifies a peripancreatic aneurysm. (B) Abdominal CT showed a 6 cm false aneurysm (arrow) arising from the SMA. (C) The angiography showed an SMA aneurysm with two floating micro-coils deployed in a failed coil angioembolization. (D) After ligating the proximal feeding vessel of the aneurysm, we incised the anterior wall of the false aneurysm. We sutured the orifice of the distal feeding vessel from the inner lumen of the aneurysm to stop backflow bleeding.

A 65-year-old man with alcohol-related chronic pancreatitis presented with jaundice for 2 weeks. Laboratory data showed obstructive jaundice (total bilirubin 196.35 μmol/l, direct bilirubin 125.71 μmol/l, alkaline phosphatase 454 IU/l, γ- Glutamyl transferase 645 IU/l, lipase 2 009 U/l). Abdominal sonography revealed a peripancreatic aneurysm with a ‘ying-yang’ sign (Fig. 4A). Abdominal CT showed a 6 cm false aneurysm at the pancreatic head, compressing the distal common bile duct with a dilated extra-hepatic biliary tract and a distended gallbladder (Fig. 4B).

The endovascular operator tried detachable coils of different sizes to treat the aneurysm but failed due to coil migration (Fig. 4C). A covered stent cannot be applied due to the risk of losing the nearby SMA branches. Thus, a salvage operation was performed by ligating the proximal feeding vessel from SMA, partial aneurysmectomy, and sealing the orifice of the distal feeding vessels from the intraluminal space of the aneurysm (Fig. 4D). The stenosis of the common bile duct resolved smoothly after excising the aneurysm and placing a temporal biliary tree internal stent for 4 months. No more recurrent aneurysms were noticed at follow-up CT in the postoperative 24 months.

### Case_4- Dorsal pancreatic artery false aneurysm

**Figure 5:**
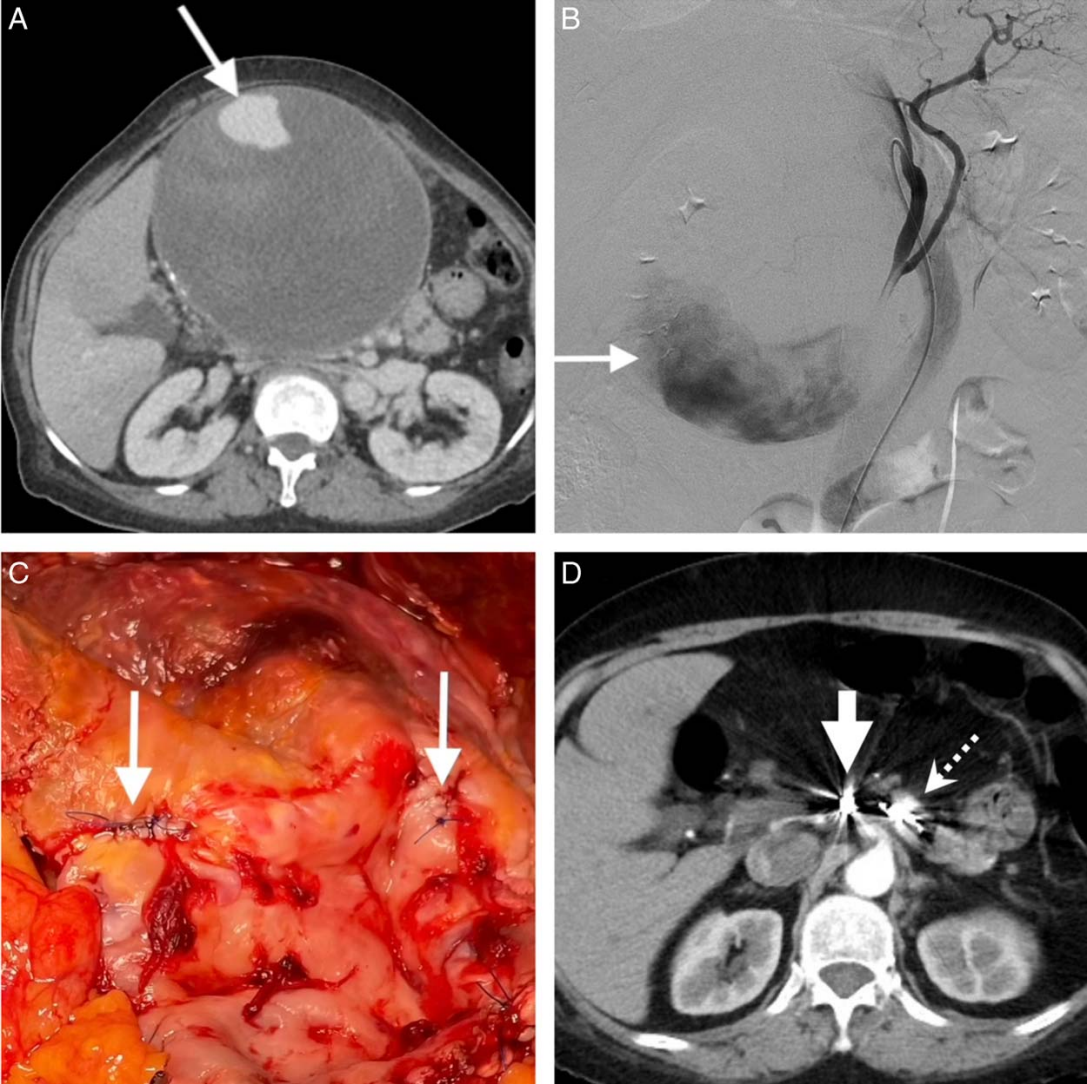
Dorsal pancreatic artery false aneurysm (A) Abdominal CT showed a peripancreatic false aneurysm about 160 mm (arrow) with intraluminal thrombosis. (B) Angiography confirmed an aneurysm from the dorsal pancreatic artery (arrow), so we performed coil angioembolization via the splenic artery but got coil migration to the distal splenic artery. (C) We offered a salvage surgery for partial aneurysmectomy and direct suturing of the feeding vessels’ orifice from the aneurysm’s inner lumen (arrow). (D) Postoperative CT showed complete resolution of the giant aneurysm with the remaining coil at the dorsal pancreatic artery (large arrow) and the migrated coil at the distal splenic artery (small arrow).

A 74-year-old woman without an underlying disease presented with periumbilical pain for 2 months. Within 2 months, she had inappetence, easy satiety, and a body weight loss of 4 kg. Abdominal CT showed a peripancreatic false aneurysm 16 cm in diameter with intraluminal partial thrombosis that caused external compression to the stomach and duodenum (Fig. 5A, B). Angiography confirmed the aneurysm arising from the dorsal pancreatic artery and occluded it proximally by coil angioembolization without N-butyl cyanoacrylate. Due to high intraluminal vascular flow, a coil migration to the distal splenic artery occurred. Considering the incompletely resolved postembolized mass effect and incomplete exclusion of the aneurysm, a salvage surgery was performed for partial aneurysmectomy and direct suturing of the orifice of feeding vessels from the intraluminal space of the aneurysm (Fig. 5C). The patient got complete resolution of the compression signs and was discharged uneventfully on postoperative day 7. No more recurrent aneurysm was observed in the postoperative 22 months (Fig. 5D).

### Case_5-celiac artery dissecting aneurysm rupture

**Figure 6:**
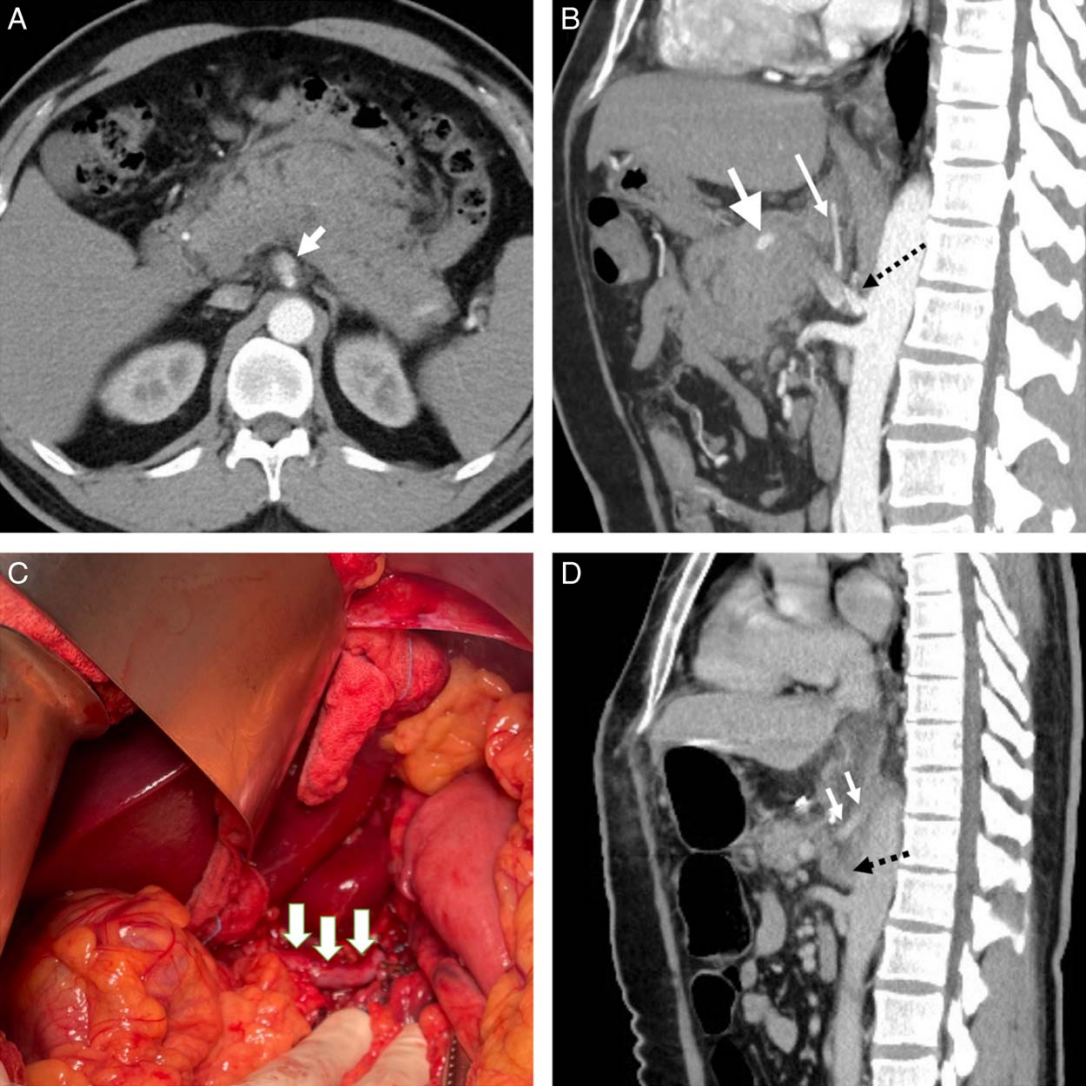
Celiac trunk dissecting aneurysm (A) Abdominal CT showed a celiac artery dissecting aneurysm about 12 mm (arrow) with rupture and extensive hematoma around the celiac trunk. (B) CT showed a dissecting aneurysm from the celiac trunk with an intima flap (black arrow). The proximal common hepatic artery (large white arrow), left gastric artery (small white arrow), and splenic artery was compressed by hematoma, causing stenotic vascular lumen and failed endovascular trials. (C) We resected the celiac false aneurysm with suturing, ligating the transected stumps of the proximal celiac artery, common hepatic artery, left gastric artery, and splenic artery. We restored the blood flow of the common hepatic artery by using a great saphenous vein as the jump graft (arrows) between the supra-celiac aorta and the common hepatic artery stump. (D) Postoperative CT showed the sutured celiac artery stump with an organized hematoma inside (black arrow) and without recurrent aneurysm. The jump graft (white arrows) was also shown.

A 47-year-old man with poorly controlled hypertension presented with sudden onset epigastralgia and upper back pain for 2 days. Abdominal CT showed celiac trunk dissection with false aneurysm rupture causing per-pancreatic hematoma and stenosis of the hepatic artery and splenic artery (Fig. 6A, B). Endovascular therapy failed due to difficulty in cannulation through the stenotic vascular lumen. A salvage surgery was performed to open the peripancreatic hematoma, and to individually control the proximal celiac trunk, splenic artery, and hepatic artery. The false aneurysm was excised, and the stumps of the proximal celiac artery, splenic artery, left gastric artery, and hepatic artery were individually sutured and ligated. Because of the large gap between the vascular stumps, the hepatic artery flow was restored by using a great saphenous vein as a jump graft between the supra-celiac aorta and the common hepatic artery (Fig. 6C). Postoperative CT showed no recurrent celiac aneurysm (Fig. 6D). The patient was discharged home uneventfully on postoperative day 7. No more recurrent aneurysm was observed in the postoperative eleven months.

## Discussion

We are the first to propose a decision-making flowchart for patients who fail endovascular trials for splanchnic aneurysms. The following strategies may decide the way of salvage surgery: aneurysmectomy along with the involved organ if the aneurysm is located within the parenchyma of solid organs, aneurysmectomy with vascular reconstruction if the aneurysm is outside the solid organ and from nonexpendable feeding vessels, and partial aneurysmectomy with direct closing of the feeding vessel orifice if the aneurysm occurs in the hostile abdomen or from expendable feeding vessels. This tailored flowchart may significantly impact future clinical practice.

### Endovascular intervention

Endovascular therapy is superior to surgical interventions in the hostile abdomen, where dense adhesion occurs due to recent surgeries or chronic inflammation (e.g., chronic pancreatitis). The commonly used endovascular therapies for the aneurysm include coil angioembolization to exclude aneurysms in expendable vessels and the covered stent to keep the patency of the nonexpendable parent vessel and the exclusion of the aneurysm concomitantly^15–17^. The feasibility of deploying a stent in splanchnic aneurysms is determined by vascular anatomy^15,17^. The need for a sufficient landing zone and the risk of blocking nearby vascular branches limit the application of covered stents in splanchnic aneurysms^17–19^.

On the other hand, the risk of coil angioembolization includes coil migration-related nontarget ischemia^20^; incomplete exclusion of the aneurysm^17^; requirement of multiple coils for sac packing in giant aneurysms; and incomplete thrombosis in ruptured aneurysms when hemorrhagic shock-related coagulopathy develops^17^. Corresponding to the current guideline, our patients received endovascular trials first, followed by salvage operations if endovascular therapy failed^5^. Insufficient landing zone for deploying the stent due to the tortuous vascular anatomy accounted for failed endovascular therapy in cases_1 and 2. Fear of losing nearby critical vascular branches after deploying the covered stent and incomplete exclusion of the aneurysms by coil angioembolization accounted for failed case_3. In case_4, a residual arterial backflow and a persistent mass effect caused by the postembolized hematoma made us have to offer a salvage surgery. In case_5, difficulty in catheter cannulation through the stenotic vascular lumen accounted for the failed endovascular trial. Therefore, we considered that the success rate of endovascular therapy in the splanchnic aneurysms near the pancreas might not be as high as in other positions because of its unique anatomic positions (i.e., tortuous, redundant, highly variable, and high-flow vascular system).

### Surgical intervention

While the endovascular approach fails, surgical intervention would be a choice. Conventionally, isolating and controlling proximal and distal vessels, followed by aneurysmectomy and vascular reconstruction, would be a standard procedure to treat the aneurysm and maintain vascular continuity^5^. Nonetheless, the procedure needs to create a proper surgical field for engaging it that may cause ungrateful bleeding and unexpected injuries to the surrounding organs, particularly in chronic pancreatitis, the most common etiology of false splanchnic aneurysms^8^. Moreover, lengthy adhesiolysis in the hostile abdomen secondary to chronic pancreatitis or recent operations is often associated with an increased risk of perioperative complications^21–23^. Hence, this might be why open surgery carries a higher complication rate than endovascular therapy in some studies^4,10^.

Since endovascular therapy is widespread, studies regarding surgical interventions for splanchnic aneurysms are relatively few. Pulli R *et al*.^1^ published their 25-year experience managing splanchnic aneurysms. As a cardiovascular surgical team, their surgical goal focused on restoring normal vascular flow. Thus, around 50% of cases received aneurysmectomy with end-to-end vascular anastomosis, even in the areas where abundant collateral flow exists (e.g., peripancreatic artery, splenic artery, or gastric arteries). Besides, they also performed aneurysmectomy with primary or patch closure or arterial ligature. However, they did not mention how to choose different surgical approaches. They reported that surgical intervention has good long-term outcomes. However, they also reported a case of mortality secondary to postoperative pancreatitis after they did an aneurysmectomy with end-to-end anastomosis for a splenic artery aneurysm. This report corresponded well with our assumption that extensive dissection in the hostile abdomen for preparing end-to-end vascular reconstruction may predispose the accidental injury and cause subsequent morbidity and mortality.

From the perspective of general surgeons, we have a slightly different philosophy for treating splanchnic aneurysms. When dissecting the vessels in the hostile abdomen is inevitable, we adopted the surgical concept learned from femoral and iliac artery aneurysm repair^24–26^. Even in a dense adhesion region, directly opening the vascular wall of the aneurysm and closing the orifice of the feeding vessels can still be done quickly. Since the collateral circulation in the peripancreatic area is abundant, proximal and distal ligation of the aneurysm can be done safely without compromising distal organ perfusion (e.g., our case_3 and 4), except in some nonexpendable vessels (e.g., SMA trunk, proper hepatic artery). Our criteria for selecting surgical approaches for splanchnic aneurysms could avoid accidental injuries caused by surgical dissection at the hostile abdomen and reduce the risk of perioperative morbidity.

### Mass effect

Either surgery or endovascular therapy as the most appropriate treatment for giant splanchnic aneurysms with associated mass effects remains undetermined^2,27–30^. Some authors preferred open surgery because postembolized thrombosis may not resolve soon^27^. Tipaldi *et al*.^2^ displayed their experience using endovascular therapy for giant splanchnic artery aneurysms in 11 cases, and one case obtained a resolution of jaundice after embolizing the aneurysm. However, considering the technically challenging and high cost, they also stated that endovascular therapy should be listed as the first-line treatment for the giant aneurysm only when the necessary expertise and resources are fully available and the patient’s hemodynamic condition is stable^2^.

Back to our experience, we think the aneurysm occurs with associated mass effects (e.g., obstructive jaundice or bowel obstruction); surgical intervention could be an alternative to provide immediate resolution of the aneurysm with associated compression. Following our strategy, incising and exploring the giant aneurysm, suturing the bleeder from the inner lumen, and emptying the contained hematoma for immediate resolution of the mass effect would be feasible and straightforward for any well-prepared surgical team. Noteworthy, because obstructive jaundice may be caused by either the aneurysm or underlying chronic pancreatitis, surgeons should rule out residual biliary stricture by intraoperative cholangiography after successful treatment for the aneurysm with associated jaundice^27^. If residual biliary stricture exists, concomitant bilioenteric bypass or postoperative endo-biliary drainage may be needed.

### Recurrent aneurysms

The residual or recurrent aneurysm after the primary surgical repair has rarely been discussed. After initial surgical repair, reoperation for a residual aneurysm is not always technically feasible^23^. Instead, endovascular therapy for the postoperative false aneurysm has been widely accepted due to its efficacy and safety^23^. Sachdev *et al*. and Spiliopoulos *et al*. highlighted a highly successful rate of repeated endovascular procedures after incomplete exclusion of splanchnic aneurysms following initial endovascular treatment^7,31^. Therefore, we consider endovascular therapy is the best choice after the incomplete exclusion of the aneurysm following open vascular surgery. However, suppose the patient suffers from an asymptomatic, small-sized aneurysm with significant medical comorbidities or a limited life expectancy. In that case, we could keep close observation rather than reinterventions unless the aneurysm shows interval growth from the surveillance imaging^5^. In our case_1, an 8-mm residual aneurysm occurred 6 months later. Given the small size (< 2 cm) of the aneurysm, asymptomatic status, and patient’s limited life expectancy due to decompensated liver cirrhosis, we adopted conservative treatment with blood pressure control and regular follow-up with serial imaging studies, which is consistent with the latest guideline of the management of splanchnic aneurysms^5^.

### Limitation

The limitation of this study includes a small number of cases and the need for long-term follow-up. Nevertheless, this study focuses on the appropriate management for splanchnic aneurysms after failed endovascular therapy and aneurysms with associated mass effects. Because both conditions are rarely discussed, our experiences with salvage surgeries for splanchnic aneurysms are still valuable, mainly providing a different perspective from general surgeons to cardiovascular specialists. Fear of blocking nearby vascular branches while deploying a covered stent for a splanchnic aneurysm was a significant cause of failed endovascular trials in this case series. However, since the splanchnic vascular system has large collateral arcades, blocking some branches by a covered stent might not be clinically relevant. Future prospective studies are warranted to answer this question.

## Conclusion

Selected true or all false splanchnic aneurysms warrant timely intervention. Surgical management is a feasible, effective, and safe alternative for splanchnic aneurysms after failed endovascular therapy or with associated mass effects.

## Ethical approval

By China Medical University Hospital institutional review board (IRB) (IRB no.: CMUH110-REC1-182) on 3 January 2022.

## Source of funding

This study was supported by China Medical University Hospital (Grant No.: DMR-110-044), Taiwan.

## Author contribution

Y.-C.L., T.-C.L., and C.-T.L contributed equally as co-first authors and were responsible for drafting the manuscript and data collection; L.-B.J., H.-R.Y., C.-H.H., and W.-C.L. supervised the study and gave professional suggestions about treatment; C.-C.Y. and C.-F.W. contributed equally as co-corresponding authors and are responsible for the study concept and design, analysis, and interpretation of data, obtaining funding.

## Conflicts of interest disclosure

The authors declare no competing financial, professional, or personal conflicts. No study sponsor is involved in the study design, data collection, analysis, or interpretation.

## Research registration unique identifying number (UIN)

The study were registered in a publicly accessible database (Registered ClinicalTrials.gov ID: NCT05727956).

## Guarantor

Chun-Chieh Yeh, one of the corresponding authors.

## Data availability statement

All data generated or analyzed during this study are included in this published article. Original material is available by contacting the corresponding author (Dr. Chun-Chieh Yeh) and can be requested by researchers who are at least getting approval from the local IRB committee.

## Supplementary Material
