# Supplementary material for: Salvage surgeries for splanchnic artery aneurysms after failed endovascular therapy: case series
Source: Int J Surg. 2023 May 18;109(7):1842–51. doi: 10.1097/JS9.0000000000000442 (PMC10389617; doi:10.1097/JS9.0000000000000442)
Supplement: Supplementary file 2 [file js9-109-1842-s002.pdf]

SDC, Table 1. summary of splanchnic aneurysms treated by endovascular therapies (2019-2022, N=73 cases)

**Location of the aneurysm**	**Superior mesenteric artery**	**Hepatic artery**	**Renal artery**	**Splenic artery**	**Gastroduodenal artery**	**Celiac trunk**	**Other visceral arteries**
**Success after endovascular treatment (N=60 cases)**	5#	13	19	13	4	0	6 (2#* right gastroepiploic a.; 2*ileocecal a.; 2* left gastric a.)
**Procedure-related complications**	0	1*liver failure after life-saving TAE for uncontrolled bleeding in a liver transplantation recipient that was rescued by salvage re-do liver transplantation but failed	0	2*splenic abscess after TAE that were successfully treated by percutaneous drainage	0	0	0
**Failure after endovascular treatment**	2	1	3	2	1	2	2 (1*right gastric a.; 1*dorsal pancreatic a.)

**(N=13 cases)**							
**The largest diameter of the aneurysm undergoing failed endo-vascular therapy**	60mm, 50mm	30mm	14mm, 26mm, 7mm	26mm, 16mm	22mm	60mm, 12mm	12mm 160mm
**Causes of failed endo-vascular therapy**	- coils migration<br>- the risk of losing nearby SMA branches after deploying a stent	- tortuous vessels<br>- the risk of occluding the right hepatic artery after deploying a stent	- coil migration<br>- technical infeasibility for catheter cannulation	- tortuous vessels<br>- hemorrhagic shock-related coagulopathy before starting TAE	- celiac trunk stenosis causing technical infeasibility for deploying a stent	- tortuous vessels and insufficient landing zone<br>- difficulty in catheter cannulation	- tortuous vessels<br>- Coils migration and persistent mass effect
**Salvage treatment or outcomes after failed**	2* partial aneurysmectomy with	1*excision of the aneurysm with end-to-end vascular reconstruction	2* nephrectomy for uncontrolled	1* loss follows, 1* initially hemorrhagic shock and coagulopathy	1*resection of the aneurysm followed by	1*partial aneurysmectomy with direct repairing;	1*partial aneurysmectomy with direct suturing of bleeders from

**endovascular therapy**	direct repairing		aneurysm bleeding 1*observation	treated by CPCR and incomplete TAE followed by multiple organ failure in a post-liver transplantation recipient	end-to-end vascular reconstruction	1*aneurysmectomy with vascular reconstruction by a jumping graft	right gastric a.; 1*partial aneurysmectomy with direct suturing of bleeders from dorsal pancreatic a.

#The numbers in the table mean the number of cases

a.: artery, TAE: trans-arterial embolization, CPCR: cardio-pulmonary-cerebral-resuscitation
